# More habitual physical activity is linked to the use of specific, more adaptive cognitive reappraisal strategies in dealing with stressful events

**DOI:** 10.1002/smi.2929

**Published:** 2020-02-09

**Authors:** Corinna M. Perchtold‐Stefan, Andreas Fink, Christian Rominger, Elisabeth M. Weiss, Ilona Papousek

**Affiliations:** ^1^ Department of Psychology University of Graz Graz Austria; ^2^ Department of Psychology University of Innsbruck Innsbruck Austria

**Keywords:** cognitive reappraisal, creativity, emotion regulation, physical activity, stress

## Abstract

Physical activity may improve stress resilience and well‐being. However, specific links to individuals' coping abilities with stressful events are sparse. This study tested whether individuals reporting more physical activity in daily life showed a higher capacity for cognitive reappraisal in dealing with potential stressors. Ninety‐eight participants reported their regular physical activity in the Freiburger Questionnaire on Physical Activity and completed a maximum performance test of their inventiveness in generating reappraisals for situations depicting real‐life stressors. The latter provides scores for overall cognitive reappraisal capacity (quantity of ideas) and preference for specific cognitive reappraisal strategies (quality of ideas; positive reinterpretation; problem‐oriented, de‐emphasizing reappraisals). Additionally, participants' anxious and depressive dispositions and general creative abilities were assessed. Results showed no association between time spent on physical activities per week and total quantity of generated reappraisal ideas. However, a higher degree of physical activity was specifically linked to a greater relative preference for the reappraisal strategy of positive reinterpretation. Opposite associations emerged for the strategy of de‐emphasizing reappraisals. The findings support the notion of more adaptive cognitive reappraisal use in more physically active individuals and may advance research on interrelationships between physical activity and cognitive and affective functions implicated in stress management.

## 1 INTRODUCTION

It is widely accepted that salutary gains of physical activity are not restricted to physical health and longevity (Lee et al., 2012) but also extend to multiple aspects of mental health (Marker, Steele, & Noser, 2018). Both these directions propose that regular physical activity may enhance a persons' ability to adaptively respond to stressors. Based on notions that physical activity may improve stressor appraisal, the present study examined whether more habitual physical activity in daily life is linked to individuals' capacity for cognitive reappraisal generation in dealing with stressful events.

### 1.1 Physical activity and stress adaptability

On the physiological level, regular exercise is commonly linked to more flexible and adaptive stress responses of the cardiovascular and cortisol system, which may represent biological adaptions in terms of lower reactivity to or faster recovery from stressful events (e.g., Blumenthal et al., 1988; Klaperski, von Dawans, Heinrichs, & Fuchs, 2013; Lackner et al., 2016). Similarly, on the psychological level, there is ample evidence that regular physical activity is related to less subjective stress (e.g., Nguyen‐Michel, Unger, Hamilton, & Spruijt‐Metz, 2006), as well as less anxiety and depression (e.g., Harvey et al., 2017; McMahon et al., 2017; Penedo & Dahn, 2005; Rebar, Duncan, Short, & Vandelanotte, 2014; Stults‐Kolehmainen & Sinha, 2014). Surprisingly, although intervention studies in both patients and nonclinical populations corroborate these beneficial effects of physical activity on stress and well‐being (e.g., Dillon, McMahon, O'Regan, & Perry, 2018; Hiles, Lamers, Milaneschi, & Penninx, 2017; Rosenbaum, Tiedemann, & Ward, 2014; Stubbs et al., 2017), much uncertainty still exists on the specific mechanisms that underpin this relationship. One hypothesis holds that physical activity increases well‐being through better coping with stressful encounters, which may include generally improved stressor appraisal or even specific styles of coping when faced with critical situations (e.g., Salmon, 2001; Sothmann, 2006; Steptoe, Kimbell, & Basford, 1998; also see Stults‐Kolehmainen & Sinha, 2014). Given the lack of research in this regard, it may be of particular interest to examine whether individuals' habitual physical activity is related to their coping abilities when facing stressors, for example, by means of cognitive reappraisal.

### 1.2 Stress coping by cognitive reappraisal

Cognitive reappraisal refers to deliberately viewing an emotionally evocative event from a different perspective and reinterpreting its meaning, thereby changing its emotional impact (e.g., Gross & John, 2003; Lazarus & Folkman, 1984). A recent meta‐analysis corroborated the power of cognitive reappraisal for dealing with stressful events, finding positive effects of reappraisal interventions on subjective stress perception (Liu, Ein, Gervasio, & Vickers, 2019, also see Webb, Miles, & Sheeran, 2012). For physical activity and stress regulation, it is proposed that a persons' physical activity may facilitate more adaptive emotion regulation in critical contexts (Bernstein, Curtiss, Wu, Barreira, & McNally, 2018; Bernstein & McNally, 2017; Shields, Matt, & Coifman, 2016). Shields et al. (2016) linked greater regular physical activity to more flexible reduction of negative emotions in response to a peer‐rejection stressor, whereas Bernstein and McNally (2017) reported that acute aerobic exercise improved stress recovery in young adults with emotion regulation difficulties. Bernstein et al. (2018) further suggested that exercise promotes greater emotional flexibility and thus enhanced adaptability when recuperating from stressful events. The only previous study on habitual physical activity and, specifically, cognitive reappraisal found a positive link between regular exercise and self‐reported success in reappraising affective pictures (Giles et al., 2017). Given these promising links with more global and indirect indices of emotion regulation, the present study scrutinized the relationship between habitual physical activity and a specific index of stress coping by reappraisal: individuals' spontaneous capacity to generate alternative appraisals for potential stressors.

### 1.3 Cognitive reappraisal capacity: Quantity and quality of reappraisals

Cognitive reappraisal capacity in the psychometric sense denotes the extent to which individuals are theoretically capable of implementing reappraisals in the face of adversity (maximum performance, Cronbach, 1970). This capacity is measured by the Reappraisal Inventiveness Test (RIT; Weber, Loureiro de Assunção, Martin, Westmeyer, & Geisler, 2014). The fluency in generating reappraisals (i.e., the quantity of ideas) in the RIT can be regarded as a direct prerequisite for effective reappraisal implementation in daily life (Papousek et al., 2017; Weber et al., 2014). Past research showed meaningful relations of reappraisal fluency with both relevant brain activation and indices of well‐being: Higher reappraisal fluency was linked to more left‐lateralized recruitment of the lateral prefrontal cortex during individuals' reappraisal of potential stressors (Papousek et al., 2017). This activation pattern also predicted practical outcomes like perceived chronic stress levels in daily life (Perchtold et al., 2018), underlining the significance of having many reappraisals readily available. Yet cognitive reappraisal is not a homogenous construct, because different types of reappraisals are often associated with different outcomes (Kalisch, Müller, & Tüscher, 2015; Shiota & Levenson, 2012; Willroth & Hilimire, 2016). Although positive (situation‐focused) reinterpretations aim at reframing negative situations in a more positive light, de‐emphasizing (self‐focused, detached) reappraisals try for a more distanced perspective of an indifferent observer or third person (Moskowitz, Hult, Bussolari, & Acree, 2009; Shiota & Levenson, 2012). Intriguingly, there are indications that positive reinterpretations are more adaptive, due to specific links with lower chronic stress experience (Perchtold et al., 2018) and greater stress resilience (e.g., Kalisch et al., 2015).

### 1.4 Physical activity and reappraisal capacity: A link through executive functions?

Cognitive reappraisal capacity is a promising candidate for scrutinizing potential effects of physical activity on well‐being, because it is strongly related to executive functions. There are consistent links between regular physical activity and domain‐general executive functions (e.g., Biddle, Ciaccioni, Thomas, & Vergeer, 2018; Daly, McMinn, & Allan, 2015; Guiney & Machado, 2013; Hamer, Terrera, & Demakakos, 2018; Lott & Jensen, 2016). In cognitive reappraisal, these general executive functions (e.g., inhibition, set‐shifting) putatively operate in an affective context, which implies domain‐specific executive demands in the cognitive control of emotion (Malooly, Genet, & Siemer, 2013; Pe, Raes, & Kuppens, 2013; Rominger et al., 2018; Weber et al., 2014). Consequently, the link between regular physical activity and domain‐general cognitive control may also extend to domain‐specific functions, as implicated in cognitive reappraisal (e.g., Rominger et al., 2018) and may thus mark a potential path through which physical activity exerts positive influence on stress regulation. Interestingly, positive reinterpretations of aversive situations may also more strongly depend on executive functions (e.g., Qi et al., 2017; Rominger et al., 2018), which suggests specific relationships of physical activity with this type of reappraisal. Given this ostensible relevance of cognitive reappraisal quality (i.e., content), the present study related physical activity to both the total quantity and the quality of cognitive reappraisal ideas in stress coping.

### 1.5 Physical activity and reappraisal capacity: The influence of creativity and affect

Potential relationships between habitual physical activity and cognitive reappraisal capacity can only be meaningfully interpreted after excluding the possibility that obtained links are due to the influence of other closely related constructs. In this study, this primarily pertains to the influence of (a) individuals' anxious and depressive affect and (b) their creative potential. Higher anxiety is consistently associated with reappraisal difficulties, and more anxious individuals are less likely to engage in physical activity (Bonnet et al., 2005; Dryman & Heimberg, 2018; Hiles et al., 2017). Similar assumptions apply to depressive symptoms, given bidirectional links with (in)effective emotion regulation and sedentary lifestyles (Joormann & Stanton, 2016; Roshanaei‐Moghaddam, Katon, & Russo, 2009). Regarding creativity, there is evidence that physical activity boosts various aspects of creative ideation (Blanchette, Ramocki, O'del, & Casey, 2005; Latorre Román, Pinillos, Pantoja Vallejo, & Berrios Aguayo, 2017; Oppezzo & Schwartz, 2014). Yet this assumption is not entirely corroborated by all studies (e.g., Colzato, Szapora Ozturk, Pannekoek, & Hommel, 2013; Frith & Loprinzi, 2018). Cognitive reappraisal capacity in the RIT is also significantly correlated with performance in traditional divergent thinking tasks (correlations of up to *r* = .61 in Weber et al., 2014; Fink et al., 2017; Rominger et al., 2018). This overlap may be due to shared cognitive demands in the production of multiple alternative solutions to an open‐ended problem, which is why cognitive reappraisal may qualify as creativity in an affective context (e.g., Fink, Perchtold, & Rominger, 2018; Fink et al., 2017). Hence, although creativity and affective dispositions do not serve as primary variables of interest in our study, they were included in statistical models to control for their influence on potential links of physical activity and cognitive reappraisal capacity.

### 1.6 The present study

**Figure 1:**
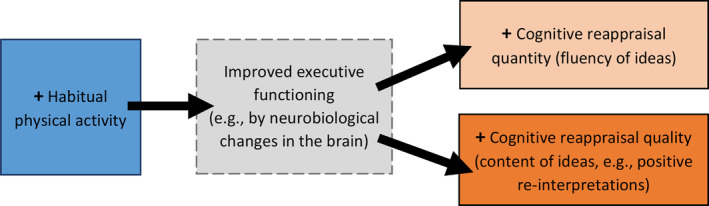
Conceptual model of the link between habitual physical activity and cognitive reappraisal capacity. The dashed frame indicates hypothesized constructs that are not directly assessed in the present study

The purpose of the present study was to determine whether individuals' regular physical activity is linked to their cognitive reappraisal capacity, beyond general creative potential, anxious and depressive dispositions. It was expected that (a) habitual physical activity may be associated with the quality of cognitive reappraisal ideas, in that physical activity might more specifically increase cognitive reappraisals more dependent on executive functions (e.g., positive reinterpretations; Biddle et al., 2018; Hamer et al., 2018; Rominger et al., 2018). Additionally, it was expected that (b) physical activity may correlate with the total quantity of cognitive reappraisal ideas, which would indicate a broader influence on cognitive reappraisal implementation (e.g., Giles et al., 2017). For a concise depiction of our conceptual model, please see Figure 1.

## 2 METHODS

### 2.1 Participants

The sample comprised 98 university students aged between 18 and 33 years (*M* = 23.06, *SD* = 3.40). Participants self‐reported their gender identification on a short demographic questionnaire (female: 54, male: 44).^1^ The required sample size was estimated a priori with G*Power (*α* = .05, 1−*β* = 0.80). Effect sizes observed in previous relevant research (*f* ^*2*^ = ~.10; Giles et al., 2017; Lott & Jensen, 2016; Shields et al., 2016) suggested a minimum of 81 participants. Individuals who reported having an acute or chronic physical or neuropsychiatric disease as well as using psychoactive medication did not participate in the study. Participants had no previous experience with the used test material. Written informed consent was obtained from all participants. The study was approved by the authorized ethics committee.

### 2.2 Physical activity

The Freiburger Questionnaire on Physical Activity (Frey, Berg, Grathwohl, & Keul, 1999) is a widely used and well‐accepted self‐assessment inventory for measuring health‐related physical activity among adults (e.g., Kleemeyer et al., 2016; Rogge et al., 2017). It consists of 12 items that cover everyday physical activities (e.g., walking to work), leisure time activities (e.g., dancing), and sports activities (e.g., swimming). Participants report the duration for each activity in minutes per week, which can be converted into metabolic equivalents (MET; Ainsworth et al., 2000). The reported hours for everyday physical activities, leisure time activities, and sports activities per week were added and used as an estimate for overall physical activity. This global score was chosen based on assumptions that individuals' cognitive reappraisal capacity is not necessarily linked to high‐intensity exercise or vigorous sports activities but to being generally more physically active in daily life (for similar arguments for mental health, see, e.g., Chekroud et al., 2018; for different accounts, see Gerber et al., 2014). Descriptive statistics for the physical activity measures and all subsequent measures are reported in Table 1. The obtained values for physical activity in this study largely correspond to values reported for younger age groups in the general population (Frey et al., 1999; also see Rogge et al., 2017). By indications of Frey and Berg (2002), our sample qualified as satisfactorily active.

**Table 1:** Descriptive statistics of all study variables

Variable	*M*	*SD*	Min	Max
**Physical activity–Time (h)**	9.24	6.40	0.50	34.70
Everyday activities	3.48	2.59	0.00	14.16
Leisure time activities	2.10	4.06	0.00	22.00
Sports activities	3.67	3.73	0.00	22.00
**Physical activity–METs**	43.15	32.43	1.50	164.20
Everyday activities	11.94	8.95	0.00	49.93
Leisure time activities	7.25	11.30	0.00	87.00
Sports activities	23.96	32.43	0.00	128.50
**Creativity**				
Verbal creativity	28.05	7.12	14.0	51.00
Figural creativity	2.04	0.79	1.00	4.00
**Affective dispositions**				
Anxious disposition	0.92	0.52	0.04	2.29
Depressive disposition	0.46	0.40	0.00	1.86
**Cognitive reappraisal capacity**				
Cognitive reappraisal quantity	21.29	5.30	9.50	39.0
Cognitive reappraisal quality				
*% positive reinterpretations*	39.18	16.66	5.26	79.55
*% de‐emphasizing*	42.43	15.49	6.00	71.88
*% problem‐oriented reappraisals*	15.79	14.71	0.00	80.00
Threat rating of RIT situations	2.95	1.01	0.50	5.50

Abbreviations: M, mean value; Max, Maximum; Min, minimum; RIT, reappraisal inventiveness test; SD, standard deviation.

### 2.3 Reappraisal inventiveness test

The RIT (Weber et al., 2014) is a maximum performance test for cognitive reappraisal ability that confronts individuals with likely stressors that may occur in their everyday lives. Participants are instructed to imagine the situation happening to them and to generate and write down as many different ways as possible to think about the situation in a way that diminishes their negative emotions. In the present study, four vignettes depicting potential stressors (de Assuncao, Golke, Geisler, & Weber, 2015; Perchtold et al., 2019) were presented one at a time on separate pages and were supplemented by a picture in order to make them more vivid. For each vignette, participants were given 20 s to imagine the situation happening to them. Then, they turned to the next page and subsequently, wrote down as many different ways to reappraise the situation with the goal to diminish stress until the allotted time of 3 min per situation had elapsed. In the night item (1), for instance, participants face the following situation: “At night, you lie alone in bed sleeping, when suddenly you are awoken by a loud noise from the next room. You get up, go into the next room and realize that the window is open.” In the other situations, individuals are confronted with walking home alone at night (2), a root canal appointment (3), and a smoke alarm going off at the neighbours (4). In line with the standard procedure of the RIT, all participants received all items in the same order (1–4). Participants' responses to the RIT items were used for the assessment of behavioural measures of their reappraisal inventiveness. Following the scoring procedure of the RIT and previous relevant research (Fink et al., 2017; Papousek et al., 2017; Perchtold et al., 2018, 2019; Rominger et al., 2018; Weber et al., 2014), RIT fluency was used as an index of quantity of reappraisal ideas, calculated as the total number of generated nonidentical reappraisals (*α* = .94). The fluency index was independently rated by two experienced researchers, with a resulting intraclass correlation (ICC) of .99 (see previous satisfying interrater reliabilities of ICC = .90 to .99 in Papousek et al., 2017; Perchtold et al., 2019; Weber et al., 2014). To gain an index of quality, reappraisal were categorized according to the category scheme of the RIT (Weber et al., 2014), which allows to categorize reappraisal ideas according to content. These reappraisal categories are positive reinterpretation (generating positive aspects; ICC = .98), de‐emphasizing (trivializing the impact of the situation; ICC = .96), and problem orientation (finding ways to reduce harm; ICC = .98). Reappraisal ideas that qualified as reappraisal of physical arousal (symptom reinterpretation) and those not matching the predefined categories were excluded from analyses due to lack of answers generated by the participants. For the analyses, the number of reappraisal ideas from eligible categories was divided by overall fluency of ideas in order to gain indices for individuals' relative preferences for a specific strategy (i.e., percentage scores). After completing the RIT, participants rated the extent of threat they would experience when confronted with the depicted situations (7‐point scales ranging from 0 [*not at all threatening*] to 6 [*very threatening*]. See Table 1 for descriptive statistics for all reappraisal measures.

### 2.4 Creativity

For the measurement of verbal creativity, the verbal imagination subscales of the well‐established German cognitive ability test (“Berliner Intelligenz‐Struktur‐Test”; Jäger, Süß, & Beauducel, 1997) were administered. Participants completed four different subtests that required them to produce and write down as many different ideas as possible in a limited amount of time (ranging between 2 and 2.5 min). Verbal creativity was scored by two trained raters. A total verbal creativity score was computed by adding the number of generated ideas in each test (see Jäger et al., 1997). Interrater reliability was excellent (ICC = .99). Creative potential in the figural domain was assessed using the Test for Creative Thinking–Drawing Production (Urban & Jellen, 1996). In the Test for Creative Thinking–Drawing Production, individuals are presented a test sheet showing six geometrical fragments and are instructed to finish the drawing in any way they like and to provide a title for the drawing within a time span of 15 min. Individuals' drawings were rated for originality by four independent raters (4‐point Likert scale ranging from 1 [*not original*] to 4 [*very original*]), which constitutes a common approach in creativity research (cf. Consensual Assessment Technique; Amabile, 1982). The originality ratings showed high interrater reliability (ICC = .85). Mean drawing time was *M* = 6.52 min (*SD* = 2.52).

### 2.5 Anxious and depressive disposition

The Personality Inventory for DSM‐5 (PID‐5, German version, Zimmermann et al., 2014) was used to obtain measures of individuals' anxious and depressive dispositions. The PID‐5 is a 220‐item questionnaire assessing maladaptive personality traits according to the DSM‐5 trait model (Section III, Emerging Measures and Models, Criterion B; Krueger, Derringer, Markon, Watson, & Skodol, 2012; Krueger & Markon, 2014). Items are scored on a 4‐point Likert scale, ranging from 0 (*very false*) to 3 (*very true*). Domain and facet scores show adequate variability also in nonclinical community and student samples with scores in the lower ranges of the scales (e.g., Bastiaens, Smits, De Hert, Vanwalleghem, & Claes, 2016; Papousek et al., 2018). The PID‐5 domain Negative Affectivity, composed of the facets Emotional Lability (seven items), Anxiousness (nine items), and Separation Insecurity (seven items) was used as a proxy of an individual's anxious disposition (*α* = .78). The PID‐5 facet of Depressivity (14 items) was used as a proxy of an individual's depressive disposition (α = .87).

### 2.6 Procedure

Testing took place in one session, in groups of three to seven participants. After general instructions, participants completed the RIT (~15 min), followed by the figural and then verbal creativity test (~15 min), as well as the physical activity questionnaire (~5 min). Finally, participants completed the entire PID‐5 (~25 min); however, only anxious and depressive disposition were analysed for the purpose of the present study. In total, this procedure lasted about 60 min.

### 2.7 Statistical analysis

The research questions were tested using four two‐step hierarchical multiple regression analyses, one with the total quantity of generated reappraisals and three with the relative use of one of the reappraisal substrategies (problem‐solving, positive reinterpretation, de‐emphasizing) as the dependent variable. In the first step, the two facets of general creative potential (verbal creativity, figural creativity) and anxious and depressive disposition were entered; in the second step, the average time of physical activity per week was added. The applied hierarchical regression approach allowed to examine whether individual differences in physical activity explained a significant amount of variance in different facets of individuals' cognitive reappraisal ideas over and above the variance afforded by individual differences in general creative potential and anxious and depressive disposition. Although our study focused on time individuals' spend on physical activities and not activity intensity, analogous regression analyses were run using METs to predict quantity and quality of generated reappraisals. The statistical assumptions for the model (i.e., ratio of cases to independent variables, normality, independence of errors, homoscedasticity, linearity, and absence of multicollinearity) were met. A significance level of *p* < .05 (two‐tailed) was used. Regression models were also run using gender or age as a covariate (first step), as well as a respective interaction term with amount of physical activity (second step), to test for potential influences on the proposed relationships. Finally, as an exploratory analysis, regression models were run separately for the physical activity subscales of everyday physical activity, leisure time activity, and sports activities, to explore whether potential correlations with reappraisal capacity were driven by time spent on a specific type of physical activity. All analyses were calculated by means of IBM SPSS Statistics 26 for windows.

## 3 RESULTS

Table 1 displays descriptive statistics for all study variables. As a preliminary analysis, one‐sample *t* tests were conducted to test whether participants viewed the RIT situations as potential personal stressors in everyday life. Subjective threat ratings for all RIT vignettes differed significantly from 0 (*t* values ranging from 14.57 to 20.93, all *p* values < .001), suggesting that on average, all situations depicted personally relevant stressors in real life. Situation 3 (“root canal,” *M* = 2.41; *SD* = 1.64) was perceived as significantly less threatening than Situation 1 (“window,” *M* = 3.36; *SD* = 1.59; *p* < .001), Situation 2 (“walking home,” *M* = 2.97, *SD* = 1.52; *p* = .051), and Situation 4 (“smoke alarm,” *M* = 3.06; *SD* = 1.57; *p* = .004).

### 3.1 Physical activity and total reappraisal capacity (quantity of generated reappraisals)

Verbal creativity positively correlated with cognitive reappraisal capacity (*sr* = .57, *p* < .001), whereas the contributions of figural creativity and anxious and depressive disposition were nonsignificant, *F*(4, 93) = 13.19, *p* < .001. Individuals' physical activity did not explain additional variance in the total quantity of generated reappraisals, *sr* = −.06, *p* = .505; *F*(5, 92) = 10.58, *p* < .001. See Table 2 for a detailed summary of the results.

**Table 2:** Summary of hierarchical multiple regression results for total reappraisal ability: Reappraisal ability (total fluency)

Variable		*R* ^*2*^	*r*	*p* (*r*)	*sr*	*p* (*sr*)	*p* (*F*) / (*F* change)	95% CI [LL, UL]
Step 1	Verbal creativity	.36	**.59**	<.001	**.57**	<.001		[0.31, 0.56]
	Figural creativity		.16	.122	.11	.183	<.001	[−0.38, 1.94]
	Anxious disposition		.08	.417	.07	.394		[−1.01, 2.55]
	Depressive disposition		.03	.739	−.03	.680		[−2.71, 1.77]
Step 2	Physical activity	.37	.03	.808	−.06	.505	.505	[−0.19, 0.10]

*Note*: Significant correlations are highlighted in bold.

Abbreviations: CI, confidence interval; LL, lower limit; *r*, Zero‐order correlation; *R* ^*2*^, proportions of variance explained by the model; *sr*, semipartial correlation; UL, upper limit.

### 3.2 Physical activity and specific reappraisal strategies (quality of generated reappraisals)

#### 3.2.1 Positive reinterpretation

Neither verbal nor figural creativity, nor anxious or depressive disposition were correlated with individuals' relative preference for using positive reinterpretations as a reappraisal strategy, *F*(4, 93) = 0.**72**; *p* = .581. Independently from their general creative potential, anxious and depressive disposition, more physically active individuals showed a greater preference for using positive reinterpretations, *sr* = .22, *p* = .033; *F*(5, 92) = 1.54, *p* = .186; see Table 3.

**Table 3:** Summary of hierarchical multiple regression results for the reappraisal strategy “positive re‐interpretation”: Relative use of reappraisal strategy “positive reinterpretation”

Variable		*R* ^*2*^	*r*	*p* (*r*)	*sr*	*p* (*sr*)	*p* (*F*) / (*F* change)	95% CI [LL, UL]
Step 1	Verbal creativity	.03	.10	.341	.06	.537	.581	[−0.33, 0.63]
	Figural creativity		.14	.179	.07	.489		[−2.85, 5.91]
	Anxious disposition		−.08	.418	−.02	.825		[−7.50, 6.00]
	Depressive disposition		−.01	.987	−.03	.772		[−9.75, 7.26]
Step 2	Physical activity	.08	**.26**	.010	**.22**	.033	.033	[.054, 1.14]

*Note*: Significant correlations are highlighted in bold.

Abbreviations: CI, confidence interval; LL, lower limit; *r*, Zero‐order correlation; *R* ^*2*^, proportions of variance explained by the model; *sr*, semipartial correlation; UL, upper limit.

#### 3.2.2 De‐emphasizing

Higher scores on both creativity measures (verbal: *sr* = −.24, *p* = .011; figural: *sr* = −.21, *p* = .021) were associated with lower preference for de‐emphasizing reappraisals. Relationships between anxious and depressive disposition and de‐emphasizing reappraisals were nonsignificant, *F*(4, 93) = 5.97, *p* < .001. More physically active individuals showed lower relative preference for implementing de‐emphasizing reappraisals, also when adjusting for individual differences in general creative potential and anxious and depressive disposition, *sr* = −.20, *p* = .028; *F*(5, 68) = 5.98, *p* < .001; see Table 4.

**Table 4:** Summary of hierarchical multiple regression results for the reappraisal strategy “de‐emphasizing”: Relative use of reappraisal strategy “de‐emphasizing”

Variable		*R* ^*2*^	*r*	*p* (*r*)	*sr*	*p* (*sr*)	*p* (*F*) / (*F* change)	95% CI [LL, UL]
Step 1	Verbal creativity	.20	**−.31**	.002	**−.24**	.011	<.001	[−0.93, −0.12]
	Figural creativity		**−.32**	.001	**−.21**	.021		[−8.03, −0.68]
	Anxious disposition		.19	.069	.08	.355		[−3.02, 8.33]
	Depressive disposition		−.16	.114	−.10	.295		[−10.94, 3.36]
Step 2	Physical activity	.25	**−.32**	.001	**−.20**	.028	.028	[−0.98, −0.06]

*Note*: Significant correlations are highlighted in bold.

Abbreviations: CI, confidence interval; LL, lower limit; *r*, Zero‐order correlation; *R* ^*2*^, proportions of variance explained by the model; *sr*, semipartial correlation; UL, upper limit.

#### 3.2.3 Problem‐oriented reappraisals

Individuals scoring higher on verbal and figural creativity showed a higher relative preference for problem‐oriented reappraisals at trend level (verbal: *sr* = .17, *p* = .085; figural: *sr* = .18, *p* = .069), whereas anxious and depressive disposition were not related to the implementation of this strategy, *F*(4, 93) = 2.35, *p* = .060. Individuals' physical activity did not explain additional variance in implementing problem‐oriented reappraisals, *sr* = .01, *p* = .942; *F*(5, 92) = 1.86, *p* = .109; see Table 5.

**Table 5:** Summary of hierarchical multiple regression results for the reappraisal strategy “problem‐oriented”: Relative use of reappraisal strategy “problem solving”

Variable		*R* ^*2*^	*r*	*p* (*r*)	*sr*	*p* (*sr*)	*p* (*F*) / (*F* change)	95% CI [LL, UL]
Step 1	Verbal creativity	.09	**.21**	.038	.17	.085	.060	[−0.05, 0.78]
	Figural creativity		**.22**	.028	.18	.069		[−0.29, 7.38]
	Anxious disposition		−.09	.404	−.04	.681		[−7.14, 4.68]
	Depressive disposition		.11	.288	.07	.469		[−4.72, 10.17]
Step 2	Physical activity	.09	.09	.381	.01	.942	.942	[−0.46, 0.50]

*Note*: Significant correlations are highlighted in bold.

Abbreviations: CI, confidence interval; LL, lower limit; *r*, Zero‐order correlation; *R* ^*2*^, proportions of variance explained by the model; *sr*, semipartial correlation; UL, upper limit.

Correlations among all study variables are reported in Table 6.

**Table 6:** Intercorrelations between study variables

Variable	Age	Physical activity—time	Physical activity—METs	Verbal creativity	Figural creativity	Anxious disposition	Depressive disposition	Reappraisal quantity	% problem‐oriented	% positive	% de‐emphasizing
Physical activity—time	.12	‐									
Physical activity—METs	.09	**.85**	‐								
Verbal creativity	.07	.13	.10	‐							
Figural creativity	.06	**.23**	.13	.12	‐						
Anxious disposition	−.13	**−.23**	**−.29**	.02	−.18	‐					
Depressive disposition	−.05	.06	−.07	.14	.02	−.14	‐				
Reappraisal quantity (fluency)	**.21**	.02	.00	**.58**	.16	.08	.03	‐			
% problem‐oriented	.00	.09	.19	**.21**	**.22**	−.09	.11	.03	‐		
% positive	.06	**.26**	.09	.10	.14	−.08	.00	.11	**−.52**	‐	
% de‐demphasizing	−.11	**−.32**	**−.23**	**−.31**	**−.32**	.18	−.16	−.12	**−.44**	**−.50**	‐
Subjective threat rating	−.13	**−.38**	**−.41**	.08	.04	**.33**	−.05	.15	.10	−.08	−.04

*Notes:* Significant Pearson correlations (*r*) are highlighted in bold font (*α* = .05). *N* = 98.

Abbreviation: METs, metabolic equivalents.

### 3.3 Metabolic equivalents

There were no significant associations between total energy expenditure associated with reported physical activities (METs) and total reappraisal capacity, relative preference for positive reinterpretations or problem‐oriented reappraisals. Correlations between METs and relative preference for implementing de‐emphasizing reappraisals resembled the pattern shown for activity time, but failed to reach statistical significance (*sr* = −.15, *p* = .118).

### 3.4 Supplementary analyses with gender and age

Women generated a higher quantity of cognitive reappraisals than men—women: *M* = 22.58, *SD* = 5.16; men: *M* = 19.69, *SD* = 5.09; *t*(96) = −2.77, *p* = .007—and also showed slightly higher verbal creativity than their male counterparts—women: *M* = 29.31, *SD* = 7.27; men: *M* = 26.49, *SD* = 6.71; *t*(96) = −1.98, *p* = .051. There were no gender differences in physical activity time, figural creativity, affective dispositions, or preference for reappraisal strategies (all *p* values > .12). However, physical activities reported by men indicated higher metabolic equivalents—men: *M* = 51.22; *SD* = 34.71; women: *M* = 36.58, *SD* = 29.15; *t*(96) = 2.27, *p* = .025. Age only significantly correlated with quantity of generated reappraisals, in that older participants demonstrated greater fluency in reappraisal of potential stressors (*r* = .20, *p* = .041; *p* values of all other correlations > .190). Adding either gender or age as well as their respective interactions with physical activity time to the analyses did not change the previous pattern of results, with no significant interaction effects with physical activity on cognitive reappraisal capacity (all *p* values for gender > .875, all *p* values for age > .321). No significant interaction effects were obtained when running these analyses with METs either (all *p* values for gender > .610; all *p* values for age > .160).

### 3.5 Exploratory analyses with specific types of physical activity

Additional analyses showed that significant correlations of physical activity with preference for positive reinterpretations were mainly driven by time spent on leisure time activities (*sr* = .28, *p* = .006), whereas correlations with de‐emphasizing reappraisals were mainly driven by time spent on everyday physical activities (*sr* = −.18, *p* = .055), but not sports activities (de‐emphasizing: *sr* = −.11, *p* = .234; positive: *sr* = .08, *p* = .439).

## 4 DISCUSSION

The present study examined whether a greater amount of habitual physical activity was related to individuals' capacity for generating reappraisals in the face of potential stressors. Although habitual physical activity was not associated with greater reappraisal fluency as in quantity of ideas, specific effects on the quality of reappraisals were observed: Individuals reporting more physical activity showed greater relative preference for implementing positive re‐interpretations and at the same time lesser preference for implementing de‐emphasizing reappraisals. These results suggest more specific effects of physical activity on cognitive reappraisal implementation in stressful contexts instead of a broader relationship.

This finding is relevant because in literature, positive reinterpretation is often referred to as both very adaptive and most difficult to implement. A positive appraisal style was found to predict greater stress resilience (Kalisch et al., 2015), better health behaviour in patients (Moskowitz et al., 2009), and improved mental health in older adults (particularly in context of physical illness; Nowlan, Wuthrich, & Rapee, 2015). Positive reinterpretations were also more effective in reducing negative emotional experience compared with de‐emphasizing reappraisals (Willroth & Hilimire, 2016). Notably, greater use of positive reinterpretations in the RIT previously predicted less chronic stress experience, whereas de‐emphasizing reappraisals did not (Perchtold et al., 2018). This may suggest that more physically active individuals show greater preference for implementing stress reappraisals of a putatively higher quality. Because these “higher quality” reappraisals predict greater stress resilience (Kalisch et al., 2015; Perchtold et al., 2018), they may act as an intermediary mechanism facilitating broader links between physical activity and well‐being. The link between habitual physical activity and higher quality reappraisals may be due to the seminal role of executive functions in successful cognitive reappraisal (e.g., Joormann & Vanderlind, 2014; Malooly et al., 2013; Pe et al., 2013). Besides the inhibition of prepotent negative aspects and memory updating, reappraisal warrants the ability to switch between negative and neutral or positive mental sets (Malooly et al., 2013; Papousek et al., 2017; Rominger et al., 2018). Rominger et al. (2018) found that correlations between cognitive reappraisal capacity and inhibition/shifting were mainly driven by positive reinterpretations, implying that good executive functioning may be more relevant for this particular strategy compared with others. These results match previous findings that linked a higher shifting ability to more successful reappraisal efforts in terms of greater reductions of self‐reported negative affect (Malooly et al., 2013). Moreover, studies found specific brain activation in frontal regions indicating cognitive effort during positive reinterpretation, which was absent during simple viewing of negative pictures (Moser, Hartwig, Moran, Jendrusina, & Kross, 2014) and during detached reappraisal (Qi et al., 2017). Further, Qi et al. (2017) reported that specific brain processes during detached reappraisal occurred long before they occurred during positive reappraisal, implying that detached reappraisals operate earlier and are less effortful in terms of executive functions. For the present study, the following is suggested: Because physical activity may be linked with better executive functioning, possibly through neurobiological changes in relevant brain regions like the frontal cortex (Erickson, Hillman, & Kramer, 2015; Guiney & Machado, 2013; Hamer et al., 2018), more physically active individuals may prefer “higher quality” reappraisals as they can more easily implement them in stressful contexts due to more functional inhibition/shifting (e.g., Malooly et al., 2013; Rominger et al., 2018). Stronger preference for positive reinterpretations then occurs at the expense of de‐emphasizing reappraisals. De‐emphasizing, though at times linked with greater reductions in emotional arousal (e.g., Dörfel et al., 2014; Shiota & Levenson, 2012), may constitute a quicker route in reappraising aversive situations and, thus, may not yield the same long‐term benefits suggested for positive reappraisal (Kalisch et al., 2015; Nowlan et al., 2015).

We can only speculate on specific physiological mechanisms that may facilitate positive effects of habitual physical activity on stress coping ability. Our previous arguments for a pathway through executive functioning align with assumptions that regular physical activity improves neuroplasticity in associated brain regions, which allows for more flexible behavioural adaptions in ever‐changing situations, perhaps also extending to more adaptive cognitive reappraisal (e.g., Erickson et al., 2015; Hötting & Röder, 2013). Suggested exercise‐related changes in physiology also entail reduced reactivity of the hypothalamic–pituitary–adrenal axis to stressors in general, facilitating faster stress recovery or greater resilience (e.g., Klaperski et al., 2013; Sothmann, 2006), and thereby, potentially greater coping success by reappraisal. Interestingly, Klaperski et al. (2013) suggested that significant adaptations of the hypothalamic–pituitary–adrenal response to stress may already occur at lower levels of physical activity, reporting a lower physiological response to psychosocial stress in both moderately and vigorously active young adults compared with rather sedentary ones. This aligns with our findings that greater habitual physical activity in general and not more sports activities per se indicated a more adaptive use of cognitive reappraisal strategies. However, although this may be interpreted as more physically active participants experiencing less stress during reappraisal of the RIT situations and thus excelling at higher quality reappraisals, note that the RIT does not necessarily elicit stress at the time of reappraisal. Rather, the RIT measures individuals' theoretical, brain‐based capacity to reappraise potentially relevant stressors (e.g., Perchtold et al., 2019). This cognitive capacity, for example, as expressed by greater preference for more adaptive, for example, positive reappraisal strategies may, however, delineate a crucial cognitive prerequisite for better stress coping in daily life.

Notably, the significant relationships in this study were present even when adjusting for individuals' anxious and depressive dispositions as well as general creative potential. The latter shows that the obtained findings are not simply a by‐product of relationships of physical activity with creative ideation (e.g., Latorre Román et al., 2017; Oppezzo & Schwartz, 2014), and creative ideation with cognitive reappraisal capacity (Fink et al., 2017; Rominger et al., 2018; Weber et al., 2014). Matching the reappraisal findings, in the present study, only figural creativity, but not verbal creativity, showed significant correlations with physical activity (for a similar specificity of findings, see for example, Cavallera, Boari, Labbrozzi, & Bello, 2011; Colzato et al., 2013). Although the verbal creativity subscales strongly drew on ideational fluency and flexibility (i.e., generate as many different ideas as possible), the figural creativity test was clearly not. Rather, it required participants to draw a single picture based on geometrical fragments, involving a much stronger focus on originality and quality compared with the verbal creativity scales. Thus, physical activity appears to have a stronger link to the originality/quality facet of creativity rather than to productivity/quantity. Strikingly, in both the affective (i.e., reappraisal) and the nonaffective domain (i.e., general creative potential), more pronounced links of physical activity to the quality rather than the quantity of idea generation were found.

That in the present study, cognitive reappraisal quality was associated with the amount of time spent on physical activities, but not their METs, may be interpreted in two ways. When using a physical activity measure that encompasses an extremely broad range of activities (i.e., basic activities, leisure time, sports), METs may not adequately estimate activity intensity and its relation to certain constructs. This corresponds to notions that the effect of METs on physical and mental health are specific to the context they are accrued in and that not all METs are the same or equally appropriate to estimate exercise intensity in all populations (e.g., Holtermann & Stamatakis, 2018). In support, it was demonstrated that (lack of) time spent on physical activities is linked to all cause‐mortality risk independent of daily METs (Diaz et al., 2017) and that links of physical activity and well‐being are often found for only duration and frequency of activity (e.g., Garcia, Archer, Moradi, & Andersson‐Arntén, 2012; McMahon et al., 2017). Alternatively, for positive effects on reappraisal quality in stress coping, it may simply be more relevant to frequently engage in various physical activities irrespective of their intensity, which may foster more flexible perspectives due to frequent changes in sceneries and activities. It was shown that the mild physical activity of walking significantly enhanced novel idea generation (in particular cognitively demanding ideas; Oppezzo & Schwartz, 2014). Frequent engagement in recreational sports was also linked to more elaborate creative ideas (Cavallera et al., 2011).

A few limitations need to be addressed. First, due to the correlational/cross‐sectional design of this study, causality and direction of influence cannot be directly inferred. Although the research background suggests that physical activity causally affects executive functions and stress regulation (e.g., Bernstein & McNally, 2017; Biddle et al., 2018), circular mechanisms are possible. In this regard, Giles et al. (2018) found that cognitive reappraisal during endurance exercise reduced perceived physical exertion. As a result, more successful cognitive reappraisal might increase the time individuals spend exercising. However, in the present study, effects were found for overall habitual physical activity (including everyday and leisure time activities) and were not limited to sports activities. Second, this study did not include a measure of life stress. Although it was previously shown that supposedly more adaptive reappraisal by means of more positive re‐interpretations predicted lower chronic stress experience in young women (Perchtold et al., 2018), future investigations are warranted to measure life stress to more directly demonstrate that effects of physical activity on theoretical stress coping abilities have practical consequences for how stressed individuals feel in daily life. Third, levels of physical activity were exclusively assessed by self‐report. Whereas future studies should incorporate more objective behavioural measures, our estimate of physical activity may suffice for correlational designs like the present study. Further, our conclusions are based on assumptions that positive reinterpretations in cognitive reappraisal are more adaptive than other strategies (e.g., de‐emphasizing). Yet the inherent adaptivity of a positive reappraisal style has been questioned, particularly in contexts where situations are more easily controllable or when positive reinterpretations are unrealistically optimistic (e.g., Egloff, 2015). Thus, given other settings, other reappraisal strategies might be the better choice. Lastly, though underpinned by research, our propositions regarding potential influences of executive functioning on the relationship between physical activity and cognitive reappraisal quality are to be considered preliminary until these potential pathways are examined in future studies.

Altogether, the present findings add to the growing body of research suggesting that physical activity is associated with various aspects of stress coping, in particular cognitive reappraisal strategies strongly depending on cognitive control (i.e., positive reinterpretation). In this respect, the link between physical activity and specific, more adaptive cognitive reappraisal strategies might signal a potential mechanism through which physical activity may exert positive effects on stress management and psychological health.

## CONFLICT OF INTEREST

The authors declare no conflict of interest.

## DATA ACCESSIBILITY STATEMENT

The raw data supporting the conclusions of this manuscript will be made available by the authors, without undue reservation, to any qualified researcher.

## Endnote

1 Participants indicated their gender on a binary scale (female, male). Although our demographic questionnaire did not provide the option “other” for gender identification, participants were encouraged to add any additional personal information they considered relevant at their discretion when filling in the questionnaire. All participants in this study identified as either male or female.
